# Mosquito Trilogy: Microbiota, Immunity and Pathogens, and Their Implications for the Control of Disease Transmission

**DOI:** 10.3389/fmicb.2021.630438

**Published:** 2021-04-06

**Authors:** Paolo Gabrieli, Silvia Caccia, Ilaria Varotto-Boccazzi, Irene Arnoldi, Giulia Barbieri, Francesco Comandatore, Sara Epis

**Affiliations:** ^1^Department of Biosciences and Pediatric Clinical Research Center “Romeo ed Enrica Invernizzi”, University of Milan, Milan, Italy; ^2^Department of Agricultural Sciences, University of Naples “Federico II”, Naples, Italy; ^3^Task Force on Microbiome Studies, University of Naples “Federico II”, Naples, Italy; ^4^Department of Biology and Biotechnology, University of Pavia, Pavia, Italy; ^5^“L. Sacco” Department of Biomedical and Clinical Sciences, Pediatric Clinical Research Center “Romeo ed Enrica Invernizzi”, University of Milan, Milan, Italy

**Keywords:** *Wolbachia*, vector-borne diseases, control strategies, pathogens, insects

## Abstract

In mosquitoes, the interaction between the gut microbiota, the immune system, and the pathogens that these insects transmit to humans and animals is regarded as a key component toward the development of control strategies, aimed at reducing the burden of severe diseases, such as malaria and dengue fever. Indeed, different microorganisms from the mosquito microbiota have been investigated for their ability to affect important traits of the biology of the host insect, related with its survival, development and reproduction. Furthermore, some microorganisms have been shown to modulate the immune response of mosquito females, significantly shaping their vector competence. Here, we will review current knowledge in this field, focusing on i) the complex interaction between the intestinal microbiota and mosquito females defenses, both in the gut and at humoral level; ii) how knowledge on these issues contributes to the development of novel and targeted strategies for the control of mosquito-borne diseases such as the use of paratransgenesis or taking advantage of the relationship between *Wolbachia* and mosquito hosts. We conclude by providing a brief overview of available knowledge on microbiota-immune system interplay in major insect vectors.

## General Introduction

Bloodsucking insects are important vectors of pathogens that cause a variety of severe diseases worldwide, with a strong impact on human and animal health (Lee et al., 2018; Boulanger et al., 2019). Concern about vector-borne diseases has increased in the last decade, also because of the geographical spread of several insect vectors, caused by intense trade and climate changes (de La Rocque et al., 2011; Caminade et al., 2019).

In particular, mosquitoes are major vectors of pathogens, including protozoa (e.g., *Plasmodium* spp. which causes malaria), nematodes (e.g., filariae), and viruses (e.g., dengue, chikungunya, West Nile, and Zika). Over 3,500 species of mosquitoes have been described, but only a limited number of them can function as disease vectors, and varying levels of specificity are observed for different types of pathogens. Overall, mosquito-borne pathogens are estimated to cause around 500,000 deaths each year, with billions of people exposed to the risk of contracting these infectious agents^1^.

So far, the most effective preventive strategies to limit the impact of mosquito-borne diseases have focused on controlling mosquito vector populations heavily relying on the use of insecticides and personal preventive measures, such as insecticide-treated nets (ITN) (Wangdi et al., 2018; Carnevale and Gay, 2019). For example, massive use of LLINs (long-lasting insecticidal nets, ITN with longer duration of effectiveness due to the incorporation of the insecticide into fibers during the manufacturing process) has greatly contributed to combat malaria (Carnevale and Gay, 2019). However, the efficacy of these control measures is hampered by the selection and spread of resistance (Hemingway, 2018), which is a complex phenomenon that accounts for modifications of multiple biochemical processes in mosquitoes (Hemingway, 2018; Ingham et al., 2020) or, also, for alterations of the mosquito biting behavior (e.g., shifts from an indoor- to an out-door host-seeking behavior) (Moiroux et al., 2012; Kreppel et al., 2020; Perugini et al., 2020). The massive use of insecticides raises also concerns, in relation to the impact on non-target species and the environment (Mansouri et al., 2017). Furthermore, the spread of invasive mosquito species to new areas requires constant monitoring and availability of new and alternative control strategies, considering that the control methodologies applied in the area of origin of a given species are not always suitable to be used in different countries and environmental conditions (Bellini et al., 2020).

The improvement of integrated vector control strategies, and in particular the development of novel environment-friendly insecticides and control approaches, is therefore urgent. In this context, insect microbiota already inspired the development of innovative control tools, such as the use of “symbiotic control” to target insect pests and vectors.

In this review we will focus our attention on the interactions between the microbiota and the vector host, with particular emphasis on the immune response. We will describe how this interaction shapes, at least partially, the vectorial capacity of mosquitoes; we will then describe the microbiota- and symbiont-based strategies that are used to control mosquitoes and mosquito-borne diseases, or that have been proposed but not yet applied. Finally, we will provide an overview of the current knowledge about the interaction between microorganisms and the immune system in other bloodsucking insect vectors.

## The Interplay Between Female Mosquito Immune System, Gut Microbiota and Vector Competence

The vector competence of mosquitoes is a biological trait that is influenced by multiple factors (Azar and Weaver, 2019). It is shaped, in the first instance, by the genetic variability of the immune effectors of the mosquito; for example, *thioester-containing protein 1* gene have multiple alleles that determine differences in susceptibility of *Anopheles* mosquitoes to the malaria infection (Le et al., 2012). The genomic variants of vectored pathogens or parasites can also play a major role, such as the case of the E1-226V variant of chikungunya virus that is preferentially transmitted by *Aedes albopictus* (Schuffenecker et al., 2006). Lastly, vector competence in mosquitoes can be also affected by the composition of the microbiota (Boissière et al., 2012).

Microorganisms, indeed, colonize different organs and tissues in mosquitoes, including gut, salivary glands and reproductive tissues (Segata et al., 2016; Scolari et al., 2019; Gao H. et al., 2020). They influence many aspects of the mosquito biology, including reproduction, development, adult survival and, overall, immunity (Coon et al., 2014). The main sites where cellular and humoral components of adult mosquito immunity exert their functions against invaders are the hemocoel with the circulating hemolymph, that contains the immune cells called hemocytes (Hillyer, 2010, 2016; Raddi et al., 2020), and the gut, which receives the sugar and blood meals and that hosts a major component of the insect microbiota (gut-associated microbiota).

**FIGURE 1:**
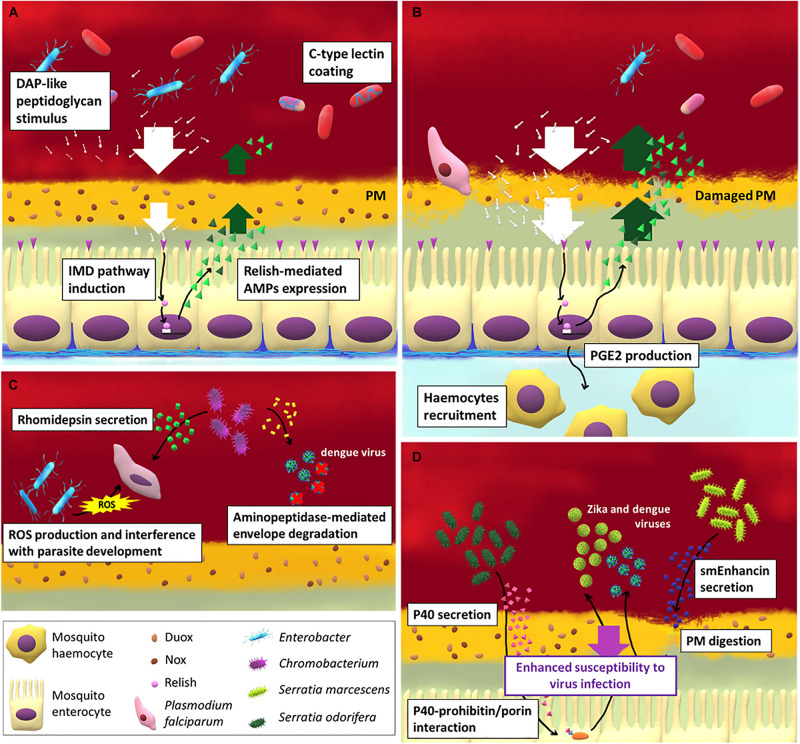
The interaction between gut immune response, microbiota and pathogens in mosquito females. **(A)** The strong increase of gut-associated microbial load after blood meal induces the activation of the IMD pathway in midgut epithelial cells and the release of antimicrobial peptides (AMPs). The contact between bacterial cells or bacterial-associated molecules (such as peptidoglycans) and epithelium is partially prevented by the peritrophic matrix (PM) which forms soon after a blood meal. Other mosquito-secreted molecules can be exploited by the bacteria as protection from AMPs, such as C-type lectins. When the PM integrity is impaired **(B)** by the action, for example, of the *Plasmodium* ookinetes, the IMD pathway is activated and hemocytes are recruited to the infection site at the base of the epithelium thanks to the release of prostaglandin E2 by midgut cells. Apart from activating vector immune response some bacterial species are able to directly limit **(C)** or to favor **(D)** pathogen and virus infection in mosquitoes.

For the purpose of this review, we will focus our attention on how bacteria interact with the gut of adult female mosquitoes and shape the immune responses after a blood meal (summarized in Figure 1). Blood meal, indeed, causes a proliferation of midgut microbiota (Gusmão et al., 2010; Kumar et al., 2010; Oliveira et al., 2011; Barletta et al., 2017) that, for instance, peaks at around 30 h after meal in *Anopheles gambiae* (Kumar et al., 2010).

Female mosquitoes acquire pathogens together with the blood meal and the microbes residing in the gut have a profound effect on the outcome of the infection (Cirimotich et al., 2011b; Dennison et al., 2014; Jupatanakul et al., 2014; Scolari et al., 2019).

For example, axenic *An. gambiae* mosquitoes are more susceptible to *Plasmodium* infection; conversely the co-feeding of a mixture of *Escherichia coli*, *Staphylococcus aureus* bacteria, and *Plasmodium falciparum* gametocytes decreases infection levels (Dong et al., 2009). Similarly, axenic *Ae. aegypti* have higher midgut dengue virus titers compared to normal septic mosquitoes (Xi et al., 2008) and some field-derived bacterial isolates affect dengue virus infection when introduced in axenic mosquitoes (Ramirez et al., 2012). Notably, the effect of microbiota on viral infection is specific and varies with the insect host and the virus: for example, it has been shown that axenic *An. gambiae* mosquitoes are less susceptible to o’nyong’nyong virus infection (Carissimo et al., 2015).

The protective role of the microbiota can be exerted by a specific class of microorganisms. It is the case of *Enterobacteriaceae* in *Anopheles mosquitoes*, which have a protective effect on *Plasmodium* infection (Cirimotich et al., 2011a; Boissière et al., 2012). In *Ae. aegypti*, different strains with different susceptibility to dengue infection harbor specific bacterial species that might be related to their vectorial capacity, with *Pedobacter* sp. and *Janthinobacterium* sp. identified only in resistant strains, while *Bacillus* sp. only in susceptible strains (Charan et al., 2013).

Physiological features and/or the genome variability of the mosquito vector can modulate vector competence in reason of their effect on the composition of gut bacteria community. The regulation of specific metabolic processes, as the branched chain amino acid degradation pathway, plays a role in the modulation of the microbial load of different *Aedes aegypti* strains (Short et al., 2017) that may in turn affect vector competence. Furthermore, genetic variation in immune genes encoding proteins with type III fibronectin domains (FN3D) in the gut correlates with interspecific variation of the load of *Serratia marcescens*, a common component of *Anopheles* gut *Enterobacteriaceae* (Stathopoulos et al., 2014). Indeed, silencing of three *FN3D* genes modulates *S. marcescens* load and alters the gut bacteria population favoring *Enterobacteriaceae* in *Anopheles* mosquitoes (Stathopoulos et al., 2014). This interaction, in turn, influences vector competence, since the abundance of *Enterobacteriaceae* in the mosquito midgut affect *Plasmodium* infection (Boissière et al., 2012).

### Humoral Immune Responses Mediated by the Gut and Interactions With the Associated Microbiota

The mosquito immune responses against infectious agents involves multiple pathways and effector molecules, which are summarized in Table 1.

**TABLE 1:** Major humoral immune pathways in mosquitoes.

Immune pathway	Pathogen/parasite	Trigger	Intracellular actors	Effectors
Toll	• Gram positive bacteria • Fungi	Binding of pathogen-derived ligands to PRRs that triggers proteolytic cleavage of the cytokine Späetzle which binds to the membrane receptor Toll	MyD88, Tube, Pelle, Relish 1, Cactus	AMPs
	• Viruses	Interaction of the virus with Späetzle or with the membrane receptor Toll		AMPs
IMD	• Gram negative bacteria	Binding of pathogen-derived ligands to PGRP membrane receptors (mainly PGRP-LC)	PGRP-LE, IMD, FADD, Dredd, Caspar, Relish 2	AMPs
	• Viruses	Binding of the virus to an unknown membrane receptor		AMPs Vago (JAK-STAT activator)
JAK-STAT	• Viruses • Parasites	Binding of Upd ligand to Domeless membrane receptor or of Vago to an unknown membrane receptor	Hop (JAK), SOCS, STAT, PIAS	AMPs Antiparasitic factors (e.g., TEP1 opsonization factor, NOS)

*The table summarizes the main features of the innate immune pathways characterized in mosquitoes (Cirimotich et al., 2009; Sim et al., 2014; Kumar et al., 2018; Mukherjee et al., 2019; Tikhe and Dimopoulos, 2021). AMP, antimicrobial peptide; PGRP, peptidoglycan recognition proteins (e.g., PGRP-LC); PRR, pattern recognition receptors (e.g., PGRP-SA, -SD); Upd, unpaired.*

The gut of mosquito females houses a wide spectrum of bacterial species, the most common of which are Gram-negative (Gendrin and Christophides, 2013; Scolari et al., 2019; Gao H. et al., 2020). Humoral responses against microbial pathogens have been deeply characterized in *Drosophila* and involve different pathways (Buchon et al., 2014; Mussabekova et al., 2017). Among them, the IMD pathway is conserved in mosquitoes (Christophides et al., 2002) and it appears to be functionally involved in antibacterial defense against both Gram-positive and Gram-negative bacteria (Meister et al., 2005; Cooper et al., 2009; Magalhaes et al., 2010; Barletta et al., 2017). In mosquito females, IMD pathway is activated in response to the proliferation of midgut microbiota that is triggered by the blood meal (Kumar et al., 2010; Barletta et al., 2017). The microbe-associated molecular pattern (MAMP) that triggers the activation of this pathway in Gram-negative bacteria is the diaminopimelic acid (DAP)-type peptidoglycan of the cell wall. In *Drosophila*, this molecule is recognized by two peptidoglycan recognition proteins (PGRP), i.e., the membrane-bound PGRP-LC in the anterior midgut and the intracellular PGRP-LE in the middle and posterior midgut (Kaneko et al., 2006; Buchon et al., 2014). Other pattern recognition proteins (PRRs) participate in the regulation of IMD pathway in a tissue specific manner: in the gut, it is positively regulated by PGRP-LA, while the amidases PGRP-LB and PGRP-SC, which cleave peptidoglycan into non-immunogenic fragments, negatively regulate the pathway (Zaidman-Rémy et al., 2006; Paredes et al., 2011; Gendrin et al., 2017). In mosquitoes, PGRP-LC is the main receptor that mediates immune response against Gram-positive and Gram-negative infections, with the isoform PGRP-LC3 recognized as key modulator of these responses at early stages of hemolymph colonization (Meister et al., 2009; Stathopoulos et al., 2014) and the isoform PGRP-LC1 having a main role in the midgut response (Rodgers et al., 2020). Similarly to *Drosophila*, PGRP-LC interacts with polymeric DAP-type peptidoglycan, while PGRP-LA and PGRP-LB positively and negatively regulate the pathway in *Anopheles* mosquitoes (Gendrin et al., 2017; Gao L. et al., 2020).

In *Drosophila*, the binding of the peptidoglycan ligand causes the dimerization of the receptor, activating an intracellular signaling cascade: the adaptor protein IMD is cleaved by the protease Dredd (Kim et al., 2014) and is rapidly ubiquitinated. This modification leads ultimately to the activation of the NF-κB transcription factor Relish, through the activity of Dredd and of the transforming growth factor β activated kinase-1 and the I-kappa B kinase complex (Paquette et al., 2010). Notably, the *An. gambiae* genome encodes two isoforms of the Relish homolog (i.e., REL-2); the short isoform, REL-2S, is involved in the response against Gram-negative bacteria, while the long isoform, REL-2F, against Gram-positives (Meister et al., 2005). It has been demonstrated that in *Anopheles dirus* REL-2F is involved in protection against both Gram-positive (with Lys-type peptidoglycan) and Gram-negative bacteria (with DAP-type peptidoglycan) (Khan et al., 2016). Relish, in turn, induces the expression of antimicrobial peptides (AMPs). These peptides have a highly conserved structure and they might exert their antimicrobial activity through peptide-lipid interaction or receptor-mediated recognition processes (Bulet et al., 1999). In mosquitoes, there are two classes of AMPs (defensins and cecropins) that have been found in many other insects, and one class, gambicins, that seems to be mosquito specific (Levashina, 2004).

Interestingly, it has been reported a direct interaction between PGRP-LD and gut-associated microbiota in *Anopheles*. Silencing of *PGRP-LD*, led to an over-activation of the immune response, leading to an over-expression of multiple AMP in *An. stephensi* prior blood feeding that causes a reduction of the bacterial load in the mosquito gut (Song et al., 2018).

A role of an immunomodulatory peroxidase (IMPer) and a dual oxidase (Duox) secreted by midgut cells in modulating gut-associated microbiota in *Anopheles* has also been described (Kajla et al., 2016) (see also section “The Interplay Between Physical Barriers Defenses in the Gut, Immune Responses, Microbiota and Implications for Vector Competence”). Indeed, when the peroxidase is silenced in *Anopheles stephensi* midgut, bacterial growth is significantly reduced by the overexpression of nitric oxide (NO) synthase gene (*NOS*), a final effector of the JAK/STAT pathway, while no significant recruitment of the classical immune pathways was observed (Kajla et al., 2016). Since NOS is a negative regulator of *Plasmodium* development (Oliveira et al., 2011), the authors suggested that the induction of the JAK/STAT pathway might be a strategy to modulate the vectorial capacity of *Anopheles* mosquitoes.

The expression of *Duox* is also regulated by a gut-membrane-associated protein, named Mesh, and the reduction of Duox activity lead to the increase of the microbiota load, suggesting that reactive oxygen species (ROS) might participate in controlling gut microbial homeostasis (Xiao et al., 2017). Notably, it has been also shown that blood meal-derived heme can decrease ROS levels in the mosquito midgut, allowing proliferation of bacteria (Oliveira et al., 2011).

The homeostatic balance governed by a tight control of both AMP transcripts and *Duox* expression is further confirmed by the effect of the mechanism exerted by the pathogenic fungus *Beauveria bassiana*: this fungus induces dysbiosis in the mosquito midgut by altering the expression of AMP transcripts and *Duox* with the secretion of the toxin oosporein, inducing bacterial growth, promoting the overgrowth of the opportunistic bacteria *S. marcescens*, which, once in the hemocoel, favors septicemia and thus the killing of mosquitoes (Wei et al., 2017).

On the other hand, the antimicrobial effect of AMPs produced by the mosquito against gut-associated microbiota is counteracted by multiple mechanisms: it has been demonstrated, for example, that the coating of bacteria with C-type lectins expressed in the mosquito midgut counteracts AMPs activity and favors gut microbiota homeostasis (Pang et al., 2016; Li et al., 2020).

The priming of the mosquito innate immune response by gut-associated microbiota can partially explain the effect of microbiota on pathogen virulence (Dong et al., 2009). In particular, some bacteria species are able to promote AMP genes expression in the gut, thus exerting a protective role against pathogens: this is the case of *Proteus* sp. in *Ae. aegypti* against dengue (Ramirez et al., 2012) and *S. marcescens* in *An. stephensi* against *Plasmodium berghei* (Bai et al., 2019).

### The Interplay Between Physical Barriers Defenses in the Gut, Immune Responses, Microbiota and Implications for Vector Competence

An important immune role in the midgut of many insects is exerted by the peritrophic matrix (PM), a gel-like structure produced by midgut (Type I PM) or cardia region (Type II PM) cells (Hegedus et al., 2009). The PM is a non-cellular, selectively permeable layer composed by a scaffold of chitin fibrils associated with glycoproteins and proteoglycans that, among other functions, represents the first line of defense providing a physical barrier between the gut flora and the epithelium (Hegedus et al., 2009). In adult mosquitoes the PM is absent but in females the distension of the midgut induced by blood ingestion triggers the formation of a thick layer of Type I PM (around 20 μm) that surrounds the blood bolus (Shao et al., 2001).

As already mentioned, during blood meal, the load of gut-associated microbiota strongly increases and, interestingly, in *Anopheles* the synthesis and the integrity of PM appears to be microbiota dependent (Rodgers et al., 2017; Song et al., 2018) as already observed for other arthropod vectors (Weiss et al., 2013; Narasimhan et al., 2014). It is unclear which signaling pathway is responsible for this phenomenon, even though a potential role for the JAK/STAT pathway, which in mosquitoes has been implicated in antiviral response (Souza-Neto et al., 2009; Jupatanakul et al., 2017), has been suggested (Rodgers et al., 2017).

The structural integrity of PM is necessary for a proper response against pathogens: for example silencing of *PGRP-LD* in *An. stephensi* causes a dysbiosis, as a consequence of the altered expression of genes that codify for structural components of the PM and thus for its integrity (Song et al., 2018). Noteworthy, the fragmentation of the PM consequent to silencing increases the vectorial potential of the mosquito thanks to the enhanced susceptibility to *P. berghei* infections (Song et al., 2018).

In *An. gambiae* mosquitoes in addition to PM, the formation of a mucin-barrier lining the epithelium has been proposed (Kumar et al., 2010). In particular, upon the increase of microbiota load induced by blood meal, IMPer and Duox enzymes are secreted and their role in a process of crosslinking between mucins that may be secreted on cell surface is proposed. Although the presence of this mucin coat has to be demonstrated yet and the mechanism by which this coat should not interfere with physiological absorption/secretion processes at microvillar surface is still unknown, this mucin-barrier may regulate the access of immune elicitors secreted by bacteria to the epithelium and, vice versa, the access of immune effectors secreted by midgut cells into the endoperitrophic space where bacteria proliferate.

When PM integrity is disrupted by ookinete invasion in malaria-vectors, the direct contact between bacteria and midgut epithelial cells primes the immune cellular response in the hemocoel (Barletta et al., 2019). Hemocytes are recruited at the midgut basal surface by the prostaglandin E2 (PGE2) that is produced and secreted by the midgut cells. Hemocytes secrete an alpha macroglobulin with a structure similar to complement C3 protein in vertebrates, named thioester-containing protein 1 (TEP1) (Blandin et al., 2004; Baxter et al., 2007), which is involved in the lysis of pathogens, mainly *Plasmodium* ookinetes. In particular, TEP1 is a complement-like opsonin that upon binding to pathogens and parasites promote their recognition by hemocytes and thus promote their phagocytosis or lysis. The link between microbiota-induced immune priming and *TEP1* expression has been further demonstrated in *An. dirus* (Wang Y. et al., 2013), showing that the microbiota participates in orchestrating the epithelial and complement-like immune responses. Hemocytes, in particular granulocytes, also participate in the phagocytosis of circulating microbes, while oenocytes are major players in the melanization response (Hillyer and Strand, 2014). The activation of this system heavily affect *Plasmodium* infection: the recruitment of hemocytes in proximity of the midgut basal surface (Barletta et al., 2019) and the production of NO (Kajla et al., 2016) leads to nitration of epithelial cells, which is required for a proper immune response against these parasites (Oliveira et al., 2012).

### Direct Effect of Gut-Associated Microbiota on Pathogen Transmission

Some gut bacterial species can affect pathogen transmission directly, without influencing the mosquito immune response. *Pseudomonas rhodesiae*, *Enterobacter ludwigii*, and *Vagococcus salmoninarium*, isolated from the *Ae. albopictus* midgut, directly inhibit La Crosse virus infection, suggesting that they may produce anti-viral molecules (Joyce et al., 2011). *Chromobacterium* sp. *Panama* strain produces an aminopeptidase that degrades the dengue virus envelope protein, reducing dengue virus infection in *Ae. aegypti* (Ramirez et al., 2014; Saraiva et al., 2018a). The same species also produces an antiparasitic protein, named rhomidepsin, which restricts *P. falciparium* infection in *An. gambiae* (Saraiva et al., 2018b). An *Enterobacter*, isolated from wild *Anopheles arabiensis* mosquito populations in Zambia, has been demonstrated to generate ROS and to interfere with *P. falciparum* development before invasion of the midgut epithelium (Cirimotich et al., 2011a).

Bacteria may also enhance the infection of vectored pathogens. *Serratia odorifera* suppresses the immune response of the host by secreting a polypeptide, P40, that interacts with the mosquito prohibitin, similar to a cysteine rich protein present in some venoms, required for virus infection in mosquitoes (Londono-Renteria et al., 2015). As a result, susceptibility of *Ae. aegypti* to both dengue and chikungunya viruses infection is enhanced (Apte-Deshpande et al., 2012, 2014). Similarly, *S. marcescens* secretes smEnhancin, a protein that digests mucins associated with the PM, making mosquitoes more susceptible to virus infection (Wu et al., 2019).

The relationship between gut-microbiota and pathogens transmitted by mosquitoes is not only one way, but it is more and more clear that pathogens can shape the microbial load in the mosquito midgut and/or the composition of the bacterial population. For example, during the pre-invasive phase, *Plasmodium vivax* significantly decrease microbial load and *16S rRNA* gene expression was not detectable before 36 h post meal, the time frame when ookinetes/early oocysts invaded the gut (Sharma et al., 2020). This suggests that *Plasmodium* can restrict bacterial growth minimizing the impact of microbiota on the mosquito immune response by out-competing the bacteria before ookinete invasion.

Finally, viral infection can shape the composition of the gut microbial community: Zika virus alters the microbiota profile in *Ae. aegypti* (Villegas et al., 2018), and chikungunya virus increases the abundance of *Enterobacteriaceae* in *Ae. albopictus* (Zouache et al., 2012).

## Microbiota-Mediated Control of Vector-Borne Diseases

**FIGURE 2:**
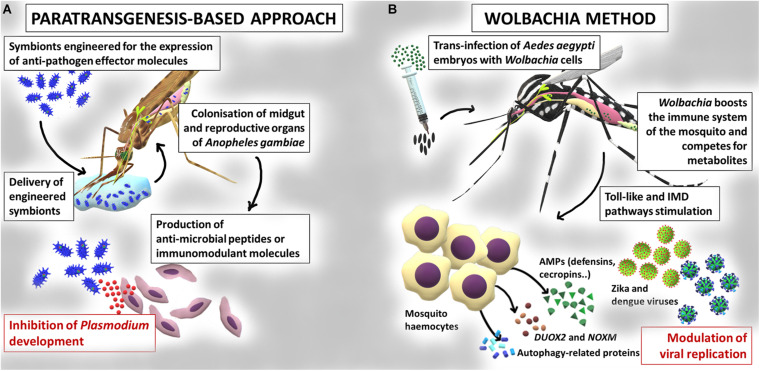
Paratransgenesis as a tool for the control of mosquito-borne diseases. **(A)** Example of application of a paratransgenesis based-approach for the control of mosquito vector competence. Engineered symbionts colonize midgut and reproductive organs of *Anopheles gambiae* mosquitoes and express anti-pathogen effector molecules, leading to the inhibition of *Plasmodium* parasite development. **(B)** Example of application of the *Wolbachia* based-approach. *Wolbachia* is artificially introduced into the *Aedes aegypti* mosquitoes; these bacteria can block development of viruses such as dengue and Zika, through priming the response of the insect immune system or competing for nutrients.

The knowledge accumulated on the interaction between insects and resident microbiota inspired the development of new strategies for the control of vector-borne diseases, since the modulation or manipulation of microbiota may have a strong impact on the host fitness and its resistance to pathogens and parasites (Gendrin et al., 2013; Gupta and Nair, 2020). The main microbiota-mediated interventions for the control of vector-borne diseases include: i) the manipulation of the symbionts for the expression of effector molecules (i.e., paratransgenesis, Wang and Jacobs-Lorena, 2017), summarized in Figure 2; ii) the introduction of microorganisms (bacteria or fungi) into the insect in order to reduce vector competence (van Tol and Dimopoulos, 2016), also outlined in Figure 2.

### Paratransgenesis for the Control of Vector-Borne Diseases

In arthropods, paratransgenesis is based on the genetic manipulation of symbionts for the production of effector molecules (e.g., antipathogens or immunomodulatory), followed by the re-introduction of the modified symbiont into the arthropod host, to reduce its vector competence (Ogaugwu and Durvasula, 2017; Wang and Jacobs-Lorena, 2017; Gao H. et al., 2020; Figure 2). The choice of a good candidate symbiont is crucial (Hoy, 2013). First, the symbiont should be stably associated with the insect vector, efficiently transmitted vertically and/or horizontally, and persist long enough to produce the effector molecules (Wilke and Marrelli, 2015). Second, the symbiont should be easily culturable and should be genetically manipulable (Wang and Jacobs-Lorena, 2017). Third, the engineered microorganism should have the same fitness of the wild type strain and should not affect the fitness of the host (van Tol and Dimopoulos, 2016). Finally, to better enhance the effect, the symbiont should secrete the antagonistic molecule to guarantee its interaction with the target pathogen (Wang and Jacobs-Lorena, 2017). Paratransgenesis was initially applied for the control of Chagas disease by exploiting the symbiont *Rhodococcus rhodnii*, engineered for the production of the AMP cecropin A in the host, the triatomine bug *Rhodnius prolixus* (Durvasula et al., 1997). Since then, several projects have explored paratrangenesis as a strategy to control malaria. In 2007, Riehle et al. (2007) engineered the bacterium *Escherichia coli* for the expression of the two anti-plasmodial molecules (i.e., salivary gland and midgut peptide 1 and the phospholipase-A2 PLA2). Although a significant inhibition of the parasite *P. berghei* development was detected, the persistence of the bacterium in the gut was very low and the expression of functional PLA2 was toxic to the bacterium (Riehle et al., 2007). The mosquito symbiotic bacteria belonging to the genera *Pantoea*, *Serratia*, and *Asaia*, have been regarded as very promising for paratransgenesis purposes. *Pantoea agglomerans* is a non-pathogenic bacterium, widespread in different mosquitoes belonging to the genus *Anopheles* and, differently from *E. coli*, can efficiently persist in the insect gut (Riehle et al., 2007). This bacterium has been engineered for the expression of five anti-*Plasmodium* factors which have determined a strong inhibition of the development of the parasite (Wang et al., 2012). *Serratia* colonizes male and female of *An. stephensi* mosquitoes with a very low fitness cost for the insect (Chiamaka et al., 2020). The release of five single effector molecules by this modified bacterium or their simultaneous expression efficiently inhibited *P. falciparum* infection in mosquitoes (Wang et al., 2017). Finally, the bacterium *Asaia*, commonly found in *Anopheles* and *Aedes* mosquitoes (Favia et al., 2007; Crotti et al., 2009) has been successfully engineered for the secretion of different effector proteins resulting in a significant inhibition of *P. berghei* development (Bongio and Lampe, 2015; Shane et al., 2018). In addition, more recently, a modified strain of the bacterium *Asaia*, able to stimulate the immune system of mosquitoes, has been proposed for the control of the heartworm *Dirofilaria immitis* (Epis et al., 2020). Examples of paratransgenic control approaches come also from the study of leishmaniases and trypanosomiasis. Engineered bacteria of the genus *Bacillus*, among others, are under study for their potential to reduce the capability of sand flies to transmit *Leishmania* (Wijerathna et al., 2020). In African trypanosomiasis, the symbiont of the genus *Sodalis* has been studied as a candidate vector to be exploited to block trypanosome transmission in the tsetse flies. Especially, attacin is a well characterized inducible immune peptide studied as an effector molecule for the engineering of *Sodalis* with specificity against some Gram-negative bacteria and protozoa (Aksoy et al., 2008).

In addition to bacteria, other microorganisms have been investigated for their potential to be exploited in paratransgenesis, in particular fungi and viruses. *Metarhizium robertsii* (previously named *M. anisopliae*), a fungus that infects several insects and proliferates in the hemolymph, was engineered to produce antimalaria effector proteins with encouraging results (Fang et al., 2011). As for viruses, densonucleosis viruses have been proposed as attractive agents for viral paratransgenesis in *Aedes* and *Anopheles* mosquitoes; Ren et al. (2008) described an efficient *An. gambiae* densovirus (*Ag*DNV) which can be potentially used for the control of malaria by transduction of anti-*Plasmodium* peptides or insect-specific toxins. The same densovirus was proposed by Suzuki et al. (2014) as over-expression system for the malaria vector *An. gambiae*. Moreover, the pathogenic *Aedes* DNV (*Ae*DNV) was manipulated to express the green fluorescent protein (Afanasiev et al., 1999) and the microRNAs that target host genes (Liu et al., 2016).

An important key point in the paratransgenic approach is the choice of the molecules with antagonistic activity against pathogens or parasites (Wang et al., 2017). While in the case of malaria parasites there are different effector molecules successfully studied and tested (Bisi and Lampe, 2011; Fang et al., 2011; Dehghan et al., 2017), in the case of viral infections the research is much more limited (Gao H. et al., 2020). Supplementary Table 1 highlights several effector molecules, including AMPs and specific single chain antibodies, currently investigated for their anti-parasite activities.

Before paratransgenesis is applied in large-scale in the field, an intermediate step is required to validate laboratory-based findings; recently, a semi-field study provided evidence for the potential capability of engineered *Asaia* bacteria to invade mosquito populations (Mancini et al., 2016). Many questions are still open about the introduction and maintenance of the engineered bacteria in mosquito populations; exploiting a bacterium that is naturally vertically and/or horizontally transmitted offers the possibility of a stably spreading the symbiont among target mosquito populations (van Tol and Dimopoulos, 2016). To date, one of the most important tools for the dissemination of engineered bacteria to mosquitoes is based on sugar baits (Lindh et al., 2006; Wang et al., 2012; Wang and Jacobs-Lorena, 2017). Furthermore, Bilgo et al. (2018) tested in a field study the attractivity and effectiveness of sugar baits as a delivery method for modified bacteria (Bilgo et al., 2018); in brief, they highlighted that Window entry trap (WET) attractive sugar bait stations are the most promising tool to introduce and spread engineered bacteria through the mosquito population. Despite these promising results and applications in semi-field condition or in the field, a real application of paratransgenesis has not yet been realized and possible disadvantages of this strategy are still to be investigated. Safety and risk assessments on humans and on non-target organisms, horizontal gene transfer, stability of the engineered symbionts in a natural habitat are some of the issues that will have to be addressed before the application (Coutinho-Abreu et al., 2010).

### Colonization of Mosquitoes With Microorganisms

The second microbiota-mediated intervention exploits the introduction of non-modified microorganisms into the insects able to impair vector competence. The impairment may occur by different mechanisms such as resource competition with the vectored pathogen or parasite, stimulation of the host immune response, reduction of host lifespan (Cirimotich et al., 2011b; Dennison et al., 2014). Different bacteria isolated from the insect gut have been studied for their capability to affect pathogen transmission. Interestingly, a recent study showed that the bacterium *S. marcescens*, isolated from the midguts of field-collected mosquitoes, could negatively affect *Plasmodium* development in *An. stephensi* mosquitoes by activating immune response and in particular modulating effector genes such as *TEP1* and *fibrinogen immunolectin 9* (Bai et al., 2019). Moreover, Cappelli et al. (2019) described the interactions between the bacteria *Asaia* and the immune system of the mosquitoes *An. stephensi*; in particular, the introduction of *Asaia* triggers mosquito immune responses, eliciting an anti-*Plasmodium* response.

To date, the most promising microbiota-mediated intervention is based on the release of *Ae. aegypti* mosquitoes infected with a *Wolbachia* strain isolated from *Drosophila melanogaster* for the control of dengue virus (Hoffmann et al., 2011; Walker et al., 2011; O’Neill, 2018; see dedicated section).

### *Wolbachia* and the Immune System of Mosquitoes

*Wolbachia* is one of the most fascinating microorganisms associated with arthropods, due to its ability to influence the reproductive biology of the hosts, their metabolism, and immunity (Werren et al., 2008). The *Wolbachia* encompasses obligate intracellular bacteria, members of the order Rickettsiales, first observed in the mosquito *Culex pipiens* by Hertig and Wolbach (1924). *Wolbachia* is widespread in insect species and populations, but patchily distributed among them. In a seminal study, insects from 65% of the examined species tested positive for *Wolbachia*, with different prevalence rates within infected species, in some cases reaching fixation (Hilgenboecker et al., 2008). Among mosquitoes, *Wolbachia* has consistently been detected in species from the genera *Culex*, *Aedes*, *Coquillettidia*, *Mansonia*, and *Uranotaenia* (Huicong et al., 2020), where it is found both in reproductive organs and somatic tissues. These localizations are coherent with the effects that *Wolbachia* has on the hosts, i.e., with its capability to influence the mosquito survival and fertility. In general, the presence of these bacteria in insects determines reproductive alterations, such as feminization of genetic males, parthenogenesis and the killing of male embryos (sex-ratio distortions) and cytoplasmic incompatibility (CI). CI provides a reproductive advantage to *Wolbachia* infected females over uninfected ones, resulting in a rapid spread of *Wolbachia* into the host population (Jiggins, 2017). CI is caused by the sperm from infected males, which is capable of reducing the fertility of uninfected females. Briefly, the molecular mechanism at the basis of CI has been recently elucidated: CI displays as embryonic death when a male expressing prophage WO genes *cifA* and *cifB* mate with an uninfected female or a female infected by an incompatible *Wolbachia* strain. In mosquito females harboring a compatible *cifA*-expressing strain rescue the embryonic development (LePage et al., 2017; Shropshire et al., 2021). *Wolbachia* has recently been detected in *Ae. aegypti* and in some species of *Anopheles* mosquitoes, although its presence is in general variable, in terms of prevalence and abundance, from species to species (Baldini et al., 2014; Balaji et al., 2019). As for the presence of *Wolbachia* in *Anopheles*, a negative correlation between *Wolbachia* infection and *Plasmodium* was observed in *An. gambiae*, in which the presence of *Wolbachia* reduces malaria transmission with effects on sporozoites (Shaw et al., 2016; Gomes et al., 2017). More recently, the description of novel *Wolbachia* strains in *Anopheles* mosquitoes was reported on two large studies in Africa (Jeffries et al., 2018; Ayala et al., 2019); in these researches the authors proved that the *Wolbachia* prevalence varied among *Anopheles* species, suggesting that the sample size can be a key factor to detect the infection. Moreover, recent papers emphasized that the evidence for the infection of *Wolbachia* in *Anopheles* mosquitoes is largely molecular, which implies that active *Wolbachia* infections had not always been discriminated from the simple presence of “traces” of *Wolbachia* or its DNA (Chrostek and Gerth, 2019; Ross et al., 2020). However, another possible explanation for the limited presence of *Wolbachia* in several *Anopheles* mosquitoes can be the preponderant role of *Asaia* bacteria in these mosquitoes (Favia et al., 2007; Chouaia et al., 2012). In fact, *Asaia* symbionts had been shown to interfere with the vertical transmission of *Wolbachia* and to negatively correlate with *Wolbachia* in mosquito reproductive tissues (Hughes et al., 2014; Rossi et al., 2015).

Prior to the observation of naturally infected individuals of *Ae. aegypti*, stable and heritable *Wolbachia* infections had been generated in laboratory colonies of this species, by embryonic microinjection of *Wolbachia* from donor species (Xi et al., 2005; Figure 2). After the release of infected mosquitoes, *Wolbachia* was then able to spread into wild *Ae. aegypti* populations, by means of the CI mechanism (Xi et al., 2005; Hoffmann et al., 2011; Nazni et al., 2019). *Wolbachia* was also stably introduced into a colony of *An. stephensi*, where the bacteria increased host resistance to *P. falciparum* (Bian et al., 2013). A similar phenomenon was observed in *Ae. aegypti* where different *Wolbachia* strains have been shown to inhibit the infection by viruses of medical relevance, such as dengue (Moreira et al., 2009; Bian et al., 2010), chikungunya (Moreira et al., 2009), West Nile (Hussain et al., 2013), Zika (Aliota et al., 2016), and filarial worms (Kambris et al., 2009).

A stable infection of *Wolbachia* into a novel mosquito host implies that this symbiont must be able to cope with the host immune system. Thus, has *Wolbachia* evolved mechanisms to suppress or stimulate the immune system of the hosts?

Actually, when *Wolbachia* bacteria infect a new host, they are able to stimulate the mosquito immune system, including the Toll and IMD pathways. In detail, Pan et al. (2018), reported that the suppression of either the IMD pathway alone or both the Toll and IMD pathways reduced *Wolbachia* load in *Ae. aegypti*; on the other hand, the activation of these pathways increased *Wolbachia* load, suggesting that host innate immunity is utilized to establish and promote this new host-microbial symbiosis. Various studies indicated that *Wolbachia*-mediated interference with pathogens is associated with a boosted immunity in mosquitoes (Kambris et al., 2009, 2010; Moreira et al., 2009; Bian et al., 2010; Hughes et al., 2011). Overexpression of AMPs, such as *defensins* and *cecropins*, and of several Toll pathway genes, is induced by *Wolbachia* in *Ae. aegypti*, providing evidence that immune activation is crucial in the inhibition of dengue infection in these mosquitoes. Comparing the transcripts of *Wolbachia*-infected *Ae. aegypti* mosquitoes with wild type mosquitoes, Pan et al. (2012) described the up-regulation of genes in the midguts of *Wolbachia*-infected mosquitoes: *defensin C*, *attacin*, *cecropin D*, *Copper superoxide dismutase*, 13 *cytochrome P450*, two putative *NADH dehydrogenase*, and three *heat-shock proteins*, *Gram-negative binding protein B1* (*GNBPB1*), *Relish-like protein 1A* (*REL1A*). Similarly, the components of the Toll pathway such as *GNBPB1*, *Spaetzle 3*, *myeloid differentiation primary response 88* and *REL1A* were also up-regulated. Moreover, they demonstrated that *Wolbachia* infection leads to an up-regulation of genes encoding a NADPH oxidase and a dual oxidase (DUOX2), which are involved in the generation of ROS. Specifically, this increased ROS level is correlated with the activation of the Toll pathway, which contributes to the production of antioxidants, defensins and cecropins (Bian et al., 2010; Luplertlop et al., 2011; Pan et al., 2012).

A recent study provided evidence for the effect of a protein of *Wolbachia* in the activation of the immune response of *Ae. aegypti* and *An. stephensi* mosquitoes, consisting in the expression of genes coding for cecropin, TEPs, leucine-rich repeat protein and CLIP-domain serine protease, plus NADPH-oxidases and NO synthase. This priming of the immune response of mosquitoes was associated with the inhibition of the development of the heartworm parasite *Dirofilaria immitis* (Epis et al., 2020; Varotto-Boccazzi et al., 2020).

Additionally, Zug and Hammerstein (2015) proposed the hypothesis that newly introduced *Wolbachia* triggers the immune response and causes oxidative stress by upregulating the expression of several immune effectors such as AMPs, autophagy-related proteins, and ROS. In *Drosophila*, a native *Wolbachia* infection increases ROS level, leading to oxidative stress, which is involved in the resistance of these flies against viral infection and replication (Wong et al., 2015). On the contrary, in *Ae. albopictus* mosquitoes, which are naturally infected by *Wolbachia*, the presence of the bacteria is not associated with oxidative stress, but with balanced redox homeostasis.

In summary, although *Wolbachia* often determines an up-regulation of mosquito immunity in newly infected hosts, immune priming is not regarded as the sole mechanism involved in the inhibition of pathogen transmission. For example, it has been proposed that competition between viruses and *Wolbachia* for intracellular cholesterol and amino acids can result in metabolite depletion and cellular stress, thus reducing viral replication (Caragata et al., 2014; Lindsey et al., 2018).

Normally, when *Wolbachia*-free insects are artificially infected with the symbionts, it is expected that an anti-microbial immune response could be triggered leading to the elimination of *Wolbachia* itself. However, *Wolbachia*, through the evasion of the AMP-based immune response or the suppression of the autophagy-associated immune defense, are able to prevent their elimination (Zug and Hammerstein, 2015). In parallel, natural selection could favor the presence of the endosymbiont *Wolbachia* improving the fitness of the insect host; indeed, other studies suggest that *Wolbachia* provides an advantage to the host in the form of metabolic provisioning (Brownlie et al., 2009; Gerth and Bleidorn, 2016). In the long term, natural selection is also expected to favor a reduction in the immune stimulating property of *Wolbachia*, with a stabilization of the association (Dedeine et al., 2003).

The artificially infection of *Aedes* mosquitoes by *Wolbachia* affects the relative abundance of resident bacteria, but not species diversity (Audsley et al., 2018), and this effect may be related to an activation of immune pathways such as Toll and IMD (Rancès et al., 2012). Interestingly, in *Anopheles* mosquitoes, there are several bacterial species that negatively correlate with *Wolbachia*; for example, Hughes et al. (2014) demonstrated that native mosquito microbiota, in particular bacteria of the genus *Asaia*, is a major barrier for the transmission of *Wolbachia*. The same observation was reported in Rossi et al., 2015, in which, a mutual exclusion or a competition between *Asaia* and *Wolbachia* has been hypothesized in anophelines thus explaining the inability of *Wolbachia* to colonize the reproductive system.

Anyhow, due to the variable influence of *Wolbachia* on the composition of mosquito microbiota, e.g., in relation with the host species, developmental stage, sampling location (Muturi et al., 2016, 2017; Straub et al., 2020), an understanding of these factors is very important before *Wolbachia* is transinfected into a new mosquito species for the control of the pathogens.

Furthermore, another crucial aspect to be investigated is the long-term phenotypic stability of artificially infected *Ae. aegypti* mosquitoes in field conditions (O’Neill, 2018). As previously described, field application of *Wolbachia*-infected *Ae. aegypti* mosquitoes for the control of mosquito-borne viruses is relatively “new”; we can expect that this system (*Wolbachia*-*Ae. aegypti*) will evolve in the coming years (Dorigatti et al., 2018). Certainly, higher efficacy strains of *Wolbachia* must be investigated and the release of mosquitoes infected by two or more strains (“superinfected”) might be proposed as an alternative strategy to manage potential reductions of the efficiency of single *Wolbachia* to interfere with pathogen transmission (Joubert et al., 2016).

## The Interaction Between Microbiota and Immune System in Other Insect Vectors

The role of microbiota in the modulation of vector immune responses and in the regulation of vector competence, has been also studied in tsetse flies (Diptera: Glossinidae), sand flies (Diptera: Psychodidae) and triatoma bugs (Hemiptera: Triatominae), major vectors of African trypanosomiasis, leishmaniases and American trypanosomiasis respectively (Cirimotich et al., 2011b; Weiss and Aksoy, 2011; Wang J. et al., 2013; Telleria et al., 2018). Indeed, the comprehension of the intimate relationship between these insect vectors and resident microbiota may be pivotal for the development of new tools to counteract the transmission and spread of diseases, such as paratransgenesis (Weiss and Aksoy, 2011).

Due to their reproduction and feeding habits, the life of the immature stages of tsetse flies is characterized by a relative sterility (Wang J. et al., 2013), since the larva develops inside the female uterus where it is fed by the maternal accessory gland (i.e., the milk gland) that produces a highly nutrient secretion. Once deposited, the larva immediately pupate, and adults, that are exclusively hematophagous, feed on sterile blood of different mammalian hosts including humans (Wang J. et al., 2013). The microbiota associated with tsetse flies is thus relatively simple compared to other insects and essentially constituted by three bacterial symbionts and a salivary-gland associated Hytrosavirus (Table 2). Moreover, the environment may marginally contribute to the establishment of gut microbiota through the ingestion of bacteria present on host skin during blood meals (Geiger et al., 2014). The obligate association with *Wiggleworthia* during larval stage is responsible for proper development of an adult functional immune system, in particular of the pathways mediating cellular responses. *Wiggleworthia*-free larvae develop into adults unable to counteract the septicemia induced by normally non-pathogenic *E. coli* due to a decrease in sessile and circulating immune cells and failure in melanization reaction (Weiss et al., 2011). Although a similar effect was observed in laboratory colonies of flies depleted of *Sodalis* and *Wolbachia*, field-flies that do not harbor these symbionts possess a functional immune system (Weiss et al., 2012). Interestingly, *Wiggleworthia* is able to trigger tsetse flies antibacterial immune responses against trypanosome by inducing the production of a peptidoglycan recognition protein (i.e., PGRP-LB) and, by the recruitment of the IMD pathway, of anti-trypanosome effector molecules (Wang et al., 2009). In addition, the competence of tsetse flies for trypanosomes has been linked to the capacity of *Wiggleworthia* to produce folate (vitamin B9) *de novo*, which thus seems to be a key metabolite for these parasites (Rio et al., 2019).

**TABLE 2:** Tsetse fly symbionts, main features of the association, and symbiont role in the modulation of host biology.

Microorganism	Features of the acquisition and association with the flies	Present in all flies?	Role in host biology	Relevant bibliography about its role in the host
*Wiggleworthia* (Fam. Enterobacteriaceae)	• Maternally transmitted bacterial endosymbiont • Localized in the cytosol of bacteriocytes adjacent to anterior midgut and also contained in milk gland secretions • Obligate mutualist	Yes	• Nutritional function (these symbionts are equipped with the biosynthetic pathways to produce vitamins essential for the host requirements) • Immunological function	Rio et al. (2019) Wang J. et al. (2013) Weiss et al. (2011, 2013)
*Sodalis* (Fam. Enterobacteriaceae)	• Maternally transmitted bacterial symbiont • Located both intra- and extra-cellularly different tissues including midgut, fat body, milk gland and salivary glands • Commensal symbiont	No	• Unknown	Toh et al. (2006) Wang J. et al. (2013) Weiss et al. (2012, 2013)
*Wolbachia* (Fam. Rickettsiaceae)	• Bacterial endosymbiont transovarically transmitted via germ line cells • Exclusively localized in germ line tissues • Parasitic symbiont	No	• Manipulation of host reproduction by different mechanisms (e.g., cytoplasmic incompatibility)	Wang J. et al. (2013) Weiss et al. (2012, 2013) Doudoumis et al. (2013)
SGHV^1^ (Fam. Hytrosaviridae)	• Horizontally transmitted during feeding • Located in salivary glands	No	• Replication causes the swelling of salivary glands (hypertrophy) • In the presence of the virus, tsetse flies may be symptomatic or asymptomatic	Wang J. et al. (2013) Kariithi et al. (2018)

*^1^Acronym for salivary gland hypertrophy virus.*

The knowledge about the interplay between microbiota and immune system in sand flies and triatome bugs is quite fragmented, although a role of intestinal microbiota in the maintenance of gut homeostasis and immune activation in these vectors has been reported (Araújo et al., 2006; Ursic-Bedoya and Lowenberger, 2007; Waniek et al., 2011; Castro et al., 2012; Diaz-Albiter et al., 2012; Vieira et al., 2015; Telleria et al., 2018).

Sand flies larvae acquire their gut microbiota from food, which is represented by soil organic matter and sand flies adults from carbohydrate-rich fluids (plant sap and aphid secretions). In addition, adult females feed on blood, principally from birds and mammals. Gut microbiota presence and composition has an impact on insect reproductive fitness (Telleria et al., 2018) and allows the activation of important immune pathways for the production of humoral effectors that allow the coexistence of insect and resident microbiota (Telleria et al., 2018). Moreover, studies on the sand fly *Lutzomyia longipalpis* have highlighted a key role of gut microbiota on vector competence for *Leishmania* (Sant’Anna et al., 2014; Kelly et al., 2017) and even that *Leishmania* protects *L. longipalpis* against bacterial infection (Diaz-Albiter et al., 2012; Sant’Anna et al., 2014). Intriguingly, recent work has demonstrated a remarkable role of *Leishmania*-infected sand fly microbiota. When regurgitated on the skin of the secondary host during bite, sand fly microbes are able to initiate an immune reaction at the bite site that positively impacts on the progression of infection (Dey et al., 2018).

The triatomine gut is a complex environment where microorganisms and parasites coexist and challenge each other in different ways (Díaz et al., 2016; de Fuentes-Vicente et al., 2018). This association has been well studied in *R. prolixus*, one of the vectors of the protozoa *Trypanosoma cruzi* (Azambuja et al., 2017). *R. prolixus* acquires enteric microbiota through horizontal transmission (i.e., by the consumption of feces of conspecifics or cannibalism, which allow the establishment of intestinal symbionts, such as *R. rhodnii* that provides vitamins to the bug) and through the skin of the animals during blood feeding, while infected blood is the source of *T. cruzi* (Azambuja et al., 2017). Although strain dependent, the capacity of the parasite to alter immune responses of the bug has been reported in different studies (Araújo et al., 2006; Ursic-Bedoya and Lowenberger, 2007; Waniek et al., 2011; Castro et al., 2012; Vieira et al., 2015). In particular, *T. cruzi* and *Trypanosoma rangeli* are able to trigger the production of immune effectors by the host (i.e., phenoloxidase and AMPs) that specifically reduce gut flora and, on the other hand, increase parasitemia (Araújo et al., 2006; Ursic-Bedoya and Lowenberger, 2007; Waniek et al., 2011; Castro et al., 2012; Vieira et al., 2015). In addition, the induction of a significant decrease of *R. rhodnii* load in the gut of *R. prolixus* infected with *T. rangeli* (but not with *T. cruzi*) has been observed (Eichler and Schaub, 2002).

## Conclusion

The manipulation of the mosquito microbiota is an emerging strategy for the control of many deadly diseases, including malaria, dengue, chikungunya, and Zika. These strategies require a deep knowledge of the mosquito immunity and of the interactions occurring between the insect immune system and the microbiota. Three main applicative approaches are under study: i) development of microbial strains that express anti-parasitic or anti-viral effector molecules; ii) development of microbial strains expressing immune-priming molecules; iii) introduction of unmodified strains with immune-priming effects in mosquitoes and/or resource competitors that ultimately limit infections in the insects. The first two approaches require the release of genetically modified organisms in the field and, therefore, further studies are needed to understand the spread and the effect of these organisms in target and non-target species. The development of strategies for a safe removal of the organisms are necessary, in the case that adverse effects will be detected during releases in the field, as already suggested for transgenic mosquitoes (Zapletal et al., 2021). The development of these multiple tools in mosquito will foster the studies in other less-studied arthropod species, which anyhow can transmit a high number of human pathogens.

## Author Contributions

PG, SC, GB, and IA reviewed the mosquito immunity, the interaction with the mosquito gut microbiota, and the interactions of microbiota with other insect species. IV-B, FC, and SE reviewed the paratransgenesis and the applied application of the studies on microbiota interaction. All authors have made a direct and intellectual contribution to the work and approved the manuscript for publication.

## Conflict of Interest

The authors declare that the research was conducted in the absence of any commercial or financial relationships that could be construed as a potential conflict of interest.

## Footnotes

1 https://www.who.int/news-room/fact-sheets/detail/vector-borne-diseases
