# Supplementary material for: Mosquito Trilogy: Microbiota, Immunity and Pathogens, and Their Implications for the Control of Disease Transmission
Source: Front Microbiol. 2021 Apr 6;12:630438. doi: 10.3389/fmicb.2021.630438 (PMC8056039; doi:10.3389/fmicb.2021.630438)
Supplement: Supplementary file 1 [file Table_1.doc]

**Supplementary Table 1: most common effector molecules for paratransgenesis**

| **Effector molecules** | **Properties** | **Microbial candidates** | **Target pathogens** | **References** |
| --- | --- | --- | --- | --- |
| Scorpine | Scorpion Pandinus imperator  venom peptide | *Pantoea agglomerans/Serratia/Asaia/ Metarhizium*  *anisopliae* | *Plasmodium*  *falciparum*  *Plasmodium*  *berghei* | Wang et al., 2012/2017; Bongio & Lampe 2015; Fang et al., 2011 |
| Shiva1 | Cecropin-like synthetic  peptide | *P. agglomerans/ Serratia* | *P. falciparum* | Wang et al., 2012/2017 |
| Cecropin A | Anopheles gambiae  cecropin A | *Rhodococcus* | *Trypanosoma cruzi* | Durvasula et al., 1997 |
| SM1 | Salivary gland and midgut  peptide 1 | *Escherichia coli* | *P. berghei* | Riehle et al., 2007 |
| [SM1]_2_ | SM1 repeat protein | *P. agglomerans* | *P. berghei* | Wang et al., 2012 |
| [SM1]_8_ | SM1 repeat protein | *M. anisopliae* | *P. falciparum* | Fang et al., 2011 |
| mPLA2 | Phospholipase A2 | *E. coli/P. agglomerans/Serratia/Asaia* | *P. falciparum*  *P. berghei* | Riehle et al., 2007; Wang et al., 2012/2017; Bongio & Lampe 2015 |
| EPIP | Enolase–plasminogen  interaction peptide | *P. agglomerans/Serratia/Asaia* | *P. falciparum*  *P. berghei* | Wang et al., 2012/2017; Bongio & Lampe 2015 |
| Pro | A chitinase propeptide | *Asaia* | *P. berghei* | Bongio & Lampe 2015 |
| Pro:EPIP | A fusion peptide composed  of a chitinase propeptide  (pro) and EPIP | *P. agglomerans/Asaia* | *P. falciparum*  *P. berghei* | Wang et al., 2012; Bongio & Lampe 2015 |
| MP2 | Midgut peptide 2 | *Serratia* | *P. falciparum* | Wang et al., 2017 |
| Pbs21scFv-Shiva1 | Single-chain immunotoxin | *P. agglomerans/E. coli/Asaia* | *P. falciparum*  *P. berghei* | Wang et al., 2012; Yoshida et al., 2001 Bongio & Lampe 2015 |
| PfNPNA-1 | Single-chain antibody | *M. anisopliae* | *P. falciparum* | Fang et al., 2011 |
| Attacin |  | *Sodalis*  *glossinidius* | *Trypanosoma brucei* | Aksoy et al., 2008 |
| WSP | Wolbachia surface protein | *Asaia* | *Dirofilaria immitis* | Epis et al., 2020 |
